# Soluble CD14 subtype (sCD14-ST) as biomarker in neonatal early-onset sepsis and late-onset sepsis: a systematic review and meta-analysis

**DOI:** 10.1186/s12865-019-0298-8

**Published:** 2019-06-03

**Authors:** Iris van Maldeghem, Charlotte M. Nusman, Douwe H. Visser

**Affiliations:** 1 Department of Pediatrics, Emma Children’s Hospital, Amsterdam UMC, University of Amsterdam, Meibergdreef 9, Amsterdam, The Netherlands; 2 Department of Neonatology, Emma Children’s Hospital, Amsterdam UMC, University of Amsterdam, Meibergdreef 9, Amsterdam, The Netherlands

**Keywords:** Neonatal sepsis, Early onset sepsis, Late onset sepsis, Soluble CD14 subtype, Presepsin

## Abstract

**Background:**

Early diagnosis of bacterial sepsis in neonates is hampered by non-specific symptoms and the lack of rapid responding laboratory measures. The biomarker soluble CD14 subtype (sCD14-ST) seems promising in the diagnostic process of neonatal sepsis. In order to evaluate the differences in diagnostic accuracy of sCD14-ST between early onset sepsis (EOS) and late onset sepsis (LOS) we assessed this systematic review and meta-analysis.

**Results:**

Twelve articles were included in the systematic review and 10 in the meta-analysis. There was a high risk of bias on patient selection, index test and/or flow and timing. The overall quality of the included studies was moderate.

At sepsis onset a consequently higher level of sCD14-ST was found in septic neonates compared to healthy controls with significant higher levels in LOS compared to EOS. In the first 24 h after sepsis onset a significant increase in pooled means of plasma sCD14-ST levels was seen in EOS (t(71.6) = 7.3, *p* < .0001) while this was not seen in LOS or healthy controls. Optimal cut-off values ranged from 305 to 672 ng/l for EOS cases versus healthy controls. The pooled sensitivity was 81% (95%CI: 0.76–0.85), the pooled specificity was 86% (0.81–0.89) with an AUC of 0.9412 (SE 0.1178). In LOS optimal cut-off values ranged from 801 to 885 ng/l with a pooled sensitivity of 81% (0.74–0.86) and a pooled specificity of 100% (0.98–1.00). An AUC and SROC was not estimable in LOS because of the low number of studies.

**Conclusions:**

sCD14-ST is a promising and rapid-responding diagnostic biomarker for EOS and LOS. The difference in pooled means between EOS and LOS underlines the importance to consider EOS and LOS as two different disease entities, requiring separate analysis in original articles and systematic reviews.

**Electronic supplementary material:**

The online version of this article (10.1186/s12865-019-0298-8) contains supplementary material, which is available to authorized users.

## Background

Worldwide bacterial sepsis is one of the major causes of neonatal morbidity and mortality and claims the lives of more than 1000 neonates every day [1]. Neonatal sepsis is commonly divided into early-onset sepsis (EOS) and late-onset sepsis (LOS). EOS reflects transplacental and ascending infections from the maternal genital tract within 72 h after birth with an incidence estimated at 0.98 per 1000 live births and [2]. LOS (≥72 h after birth) is associated with the nosocomial environment with incidence inversely associated with birth weight and gestational age [3, 4]. Rapid diagnosis of both EOS and LOS is problematic because clinical signs start subtle and look like those caused by various non-infective conditions. The golden standard diagnostic test for bacterial sepsis is microbacterial confirmation. Unfortunately, bacterial culture is time-consuming and lacks sensitivity. Therefore, neonates with risk factors for infection or clinical signs and symptoms of infection are treated with antibiotics empirically.

To avoid unnecessary treatment of non-infected neonates, an early, sensitive and specific laboratory test would be helpful to guide clinicians to decide whether or not to start antibiotics. The upcoming biomarker soluble cluster of differentiation 14 subtype (sCD14-ST), also named presepsin, could be of diagnostic value for the detection of neonatal sepsis. sCD14-ST is a soluble and truncated form of CD14, a multifunctional cell surface glycoprotein expressed by various components of the innate immunity, like monocytic and dendritic cells and neutrophils. CD14 operates as a high-affinity receptor for e.g. lipopolysaccharides (LPS) and activates the toll-like receptor 4-specific proinflammatory signaling cascade [5, 6]. Furthermore CD14 is involved in the recognition of a wide variety of other bacterial products, like peptidoglycans of gram-positive bacteria [5]. During systemic inflammation and bacterial infections circulating plasma proteases cleave the soluble fraction of CD14 and sCD14-ST is released in the circulation. In an in vitro human experimental sepsis model (challenge with LPS in a human cell line of monocytic cells, and in peripheral mononuclear cells) sCD14-ST was detected 1 h after LPS exposure and peaked at 3 h [7]. The quick detection makes sCD14-ST a potential and promising candidate bedside biomarker for neonatal sepsis. Data about the diagnostic accuracy of plasma sCD14-ST in neonatal sepsis is limited, however recently the first systematic review and meta-analysis has been published [8]. Bellos et al. focussed on diagnostic accuracy and concluded that sCD14-ST has high sensitivity and specificity for the diagnosis of neonatal sepsis (EOS and LOS together) and is superior when compared to C-reactive protein (CRP) and procalcitonin (PCT). However, EOS and LOS were analysed together while the serum levels of sCD14-ST in both disease entities seem to differ as a result of differences in blood sampling time due to a different moment of clinical presentation. In EOS an increase in sCD14-ST plasma levels is seen in the first 24 h [9], in contrast to LOS [10]. After treatment initiation sCD14-ST levels decrease in the next few days in both EOS and LOS [9–13]. In order to evaluate the differences between EOS and LOS and its influence on the diagnostic accuracy of sCD14-ST we performed a new meta-analysis of the current literature, analyzing EOS and LOS separately.

## Methods

The present study was conducted and reported according to the Preferred Reporting Items for Systematic Reviews and Meta-analyses Statement (PRISMA, see Additional file 1) [14]. The protocol of this review was published in advance on the PROSPERO database with reference number CRD42017061026.

### Literature search

A systemic literature search was performed on March 16th 2017 using the following databases and other sources: PUBMED-Medline, EMBASE, The Cochrane Library, Web of Science, Clinicaltrials.gov and WHO ICTRP Clinical Trials in Children (CTC). There were no restrictions in language and publication period. Search terms or database specific medical subject headings (MeSH terms) used were “presepsin”, “sCD14”, “sCD14-ST”, “soluble CD14” and “P-SEP”. The following keywords and MeSH terms were used to identify only studies in neonates: “child”, “Infant, Newborn”, “newborn”, “prematur”, “low birth weight”, “VLBW”, “LBW”, “infant”, “neonat”, “postmatur”, “preterm”, “new-born” and “neo-nat”. Keywords were truncated if necessary by using “*” to broaden the search (for details and logbook of the literature search see Additional file 2). During the writing process updates of the initial search were performed with relevant articles included after the initial search (last time June 4th 2018).

### Study selection

Eligibility assessment was performed in a blinded standardized manner by three reviewers. After the literature search was completed, all articles were uploaded in Rayyan and checked for duplication. Rayyan is a web and mobile app for systematic reviews which helps in the initial screening of articles based on titles and abstracts [15]. First, three independent researchers (IM, CN and DV) screened all retrieved articles on title and abstract using Rayyan application. Subsequently, all selected articles were read in full text by the three reviewers to receive the final inclusion. Any disagreement between the three reviewers regarding the eligibility of particular studies was resolved through discussion.

### Quality assessment

Two independent reviewers (IM and CN) assessed the methodological quality and the risk of bias of the included studies using the Quality Assessment of Diagnostic Accuracy Studies 2 (QUADAS-2) tool [16]. Disagreements between the two review authors about the risk of bias in particular studies were resolved through discussion and if needed involvement by the third review author (DV) was requested. Quality assessment according to the QUADAS-2 tool involved four domains; patient selection, index test, reference standard and flow and timing. In addition, applicability concerns of the first three domains was also assessed. Using the QUADAS-2 tool bias can be judged in terms of “low” or “high” risk of bias and in case there was insufficient data reported to make a judgement also as “unclear” risk.

### Data extraction

Data extraction was performed by the primary and secondary reviewer (IM and CN). Qualitative data and information was extracted from each individual study on study characteristics and study results using a structured data table. Characteristics which were obtained included study design, inclusion and exclusion criteria, number of patients, gestational age and type of measurement (including measuring instruments and time of measurement). Extracted study outcomes were reference ranges and cut-off values of sCD14-ST in both healthy and septic neonates and descriptive measures, like median, interquartile range (IQR), mean and standard deviation (SD). Components of diagnostic accuracy were also obtained and included sensitivity, specificity, area under the curve (AUC), positive predicted value (PPV), negative predicted value (NPV). EOS and LOS were analysed separately.

Most articles reported the values of sCD14-ST in ng/l as unit of measurement, but some studies reported levels in pg/ml. Since the value of both units of measurement are the same, we choose to report all values and measurements for sCD14-St in ng/l, as this is the unit most used in recent literature. In this study t = 0 represents the moment (in days) when the infant newborn is suspected of having sepsis and blood culture is taken prior to start antibiotics.

From articles in which the necessary values of sCD14-ST were only reported in graphical form, the required data were interpolated from the figures by two independent researchers.

### Data synthesis

MetaDiSc 1.4 software was used to perform the analyses of the meta-analysis [17]. When the mean and SD were reported in the article, these values were directly adapted to calculate the pooled mean and SD. When these data were not reported the estimated mean and SD were calculated based on studies of Hozo et al. and Wan et al. [18, 19], (for details see Appendix). An one-way ANOVA with post-hoc test was conducted to compare pooled means of EOS cases, LOS cases and healthy controls. An unpaired t test with Welch’s correction was performed to analyse the difference of sCD14-ST plasma levels between different time-points. The remainder of extracted data is presented in tabular or graphical form using MetaDiSc or GraphPad Prism version 7 [20]. Heterogeneity of the included studies was calculated by the inconsistency index (I-squared [21]) and is illustrated in a summary ROC plot (SROC plot) [22]. The potential presence of threshold effect was evaluated by calculating the Spearman rank correlation coefficient [17]. Publication bias was not expected to play a role since sCD14-ST is a relatively new subject of interest in scientific literature and only data for diagnostic accuracy were analysed. A funnel plot in meta-analysis of diagnostic test accuracy can be misleading and therefore was not determined [23]. In this article results of EOS, LOS and neonatal sepsis (EOS and LOS combined) will be reported separately because serum concentrations of sCD14-ST in these entities are expected to be different due to timing of blood sample collection based on clinical symptoms.

## Results

### Selection of articles

**Fig. 1:**
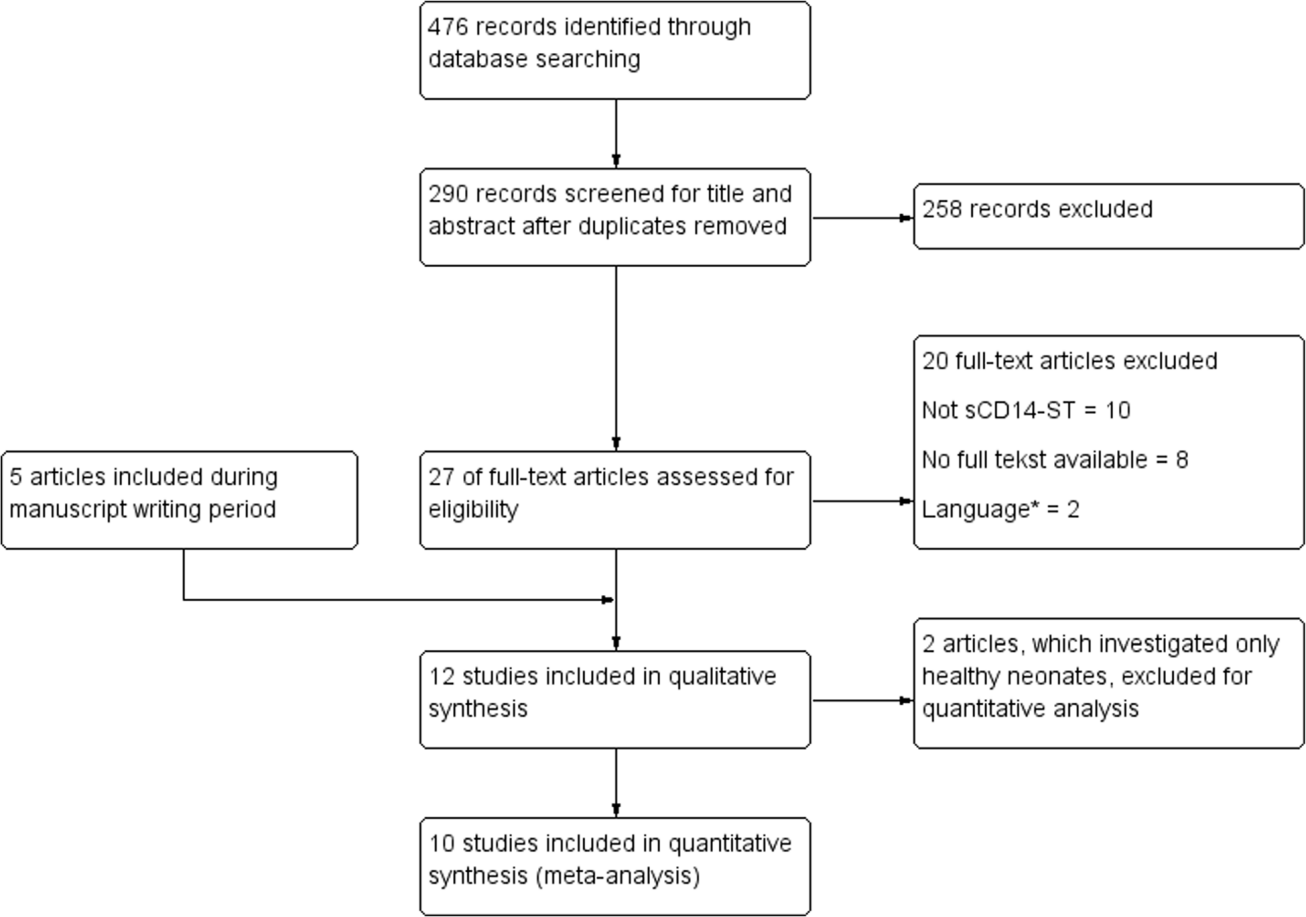
Flow diagram literature search. * Two studies were excluded during full text reading based on language (Polish and Turkish) because none of the reviewers were competent in this languages

After duplicates were removed, 290 articles remained and were screened for eligibility on title and abstract. Secondly, 263 articles were excluded because they did not match the inclusion criteria (i.e. sepsis, neonates or presepsin/sCD14-ST). The remaining 27 articles were read in full text and in total, seven articles were initially selected to get used in this systematic review. Within the data extraction and manuscript writing period three relevant articles were published and included nonetheless afterwards. As mentioned before, another meta-analysis on sCD14-ST in neonatal sepsis was published in this period [8]. In this review five articles were not found in the databases we used for our literature search. Out of those five articles, one article was not available in full text [24] and two other articles were not eligible for our review (in one article it was unclear whether they investigated EOS or LOS [25] and in one article unusual ranges of sCD14-ST values were used [26]). Therefore out of this review, two articles [27, 28] were found to be appropriate to use in our review and were included afterwards. The literature search is summarized in Fig. 1.

### Study characteristics

In total 12 studies were included for qualitative synthesis in this systematic review. An overview of the characteristics of these studies is shown in Table 1 [9–13, 27–33]. All included studies were prospective observational studies and studied septic and healthy neonates. There were two studies which only investigated healthy neonates to asses reference ranges [29, 31]. Three studies investigated neonates with EOS only [9, 11, 28], two with LOS only [10, 12] and five studies investigated both EOS and LOS [13, 27, 30, 32, 33]. All sCD14-ST measurements were done on a PATHFAST analyser, which is a non-competitive chemiluminescent enzyme immunoassay (CLEIA). However, there were two producers; Mitsubishi Chemical Medience Corporation from Tokyo, Japan and Gepa Diagnostics from Milan, Italy. All studies which included both healthy and septic neonates measured sCD14-ST at t = 0. Most studies used bacterial culture as reference standard.

**Table 1:** Overview and characteristics of included studies

Sepsis type	Author	Case definition	Cases	GA	BW	Controls	GA	BW	PATHFAST^b^	Sampling time^c^
			N	Weeks	Gram	N	Weeks	Gram		Days
EOS	Montaldo [9]	Culture positive	32	29.9 ± 1.1	1082 ± 205	38	30.2 ± 1.4	1102 ± 192	M	T = 0, ½, 1 and 2
	Ozdemir [11]	Clinical + laboratory signs	29	39.1 ± 0.9	3286 ± 461	40	39.0 ± 1.0	3379 ± 421	M	T = 0, 3 and 7
	Motalib [28]	Clinical signs	28	37.6 ± 1.7	3242 ± 269	34	38.3 ± 1.3	3198 ± 235	M	T = 0 and 7
LOS	Poggi [10]	Clinical + laboratory signs	19	25.6 ± 2.0	684 ± 215	21	28.8 ± 2.0	1021 ± 233	G	T = 0, 1, 3 and 5
	Topcuoglu [12]	Clinical signs	42	28.4 ± 2.6	1084 ± 318	40	28.9 ± 2.8	1140 ± 338	M	T = 0, 2 and 6
Controls	Mussap [29]	n/a	n/a	n/a	n/a	26	26–36	n/a	G	T = 1–7^d^
	Pugni [31]	n/a	n/a	n/a	n/a	684	≥ 24	n/a	M	T = 3–7^d^
Combined^a^	Miyosawa [32]	Culture positive + clinical signs	13	30.3 ± 6.6	1644 ± 1202	18	31.2 ± 3.4	1520 ± 605	M	T = 0, 1 and 2
	Mussap [30]	Culture positive/ clinical signs	25	35.0 (31.0–41.0)	2508 (1295–3160)	25	34.0 (29.0–36.0)	1750 (1027–3000)	G	T = 0
	Osman [27]	Clinical + laboratory signs	40	37.5 ± 1.2	2518 ± 532	15	37.8 ± 1.6	2581 ± 513	M	T = 0
	Iskandar [33]	Culture positive	35	n/a	n/a	16	n/a	n/a	M	T = 0
	Xiao [13]^e^	Culture positive	42	37.9 ± 2.4	2950 ± 639	53	38.8 ± 1.4	3094 ± 586	M	T = 0, 3 and 5
		Clinical signs	54	37.9 ± 2.6	3054 ± 563					
Total			359			1010				

*EOS* early-onset sepsis, *LOS* late-onset sepsis, ^a^studies that analyzed both EOS and LOS;

*GA* gestational age in mean ± SD or median (IQR), *BW* birth weight (mean ± SD); *n/a* not applicable

^b^*M* Mitsubishi Chemical Medience Corporation, Tokyo, Japan; *G* Gepa Diagnostics, Milan, Italy

^c^Days after admission/medical evaluation; ^d^days after birth; ^e^Only blood culture confirmed cases and healthy controls used

### Quality of studies

**Fig. 2:**
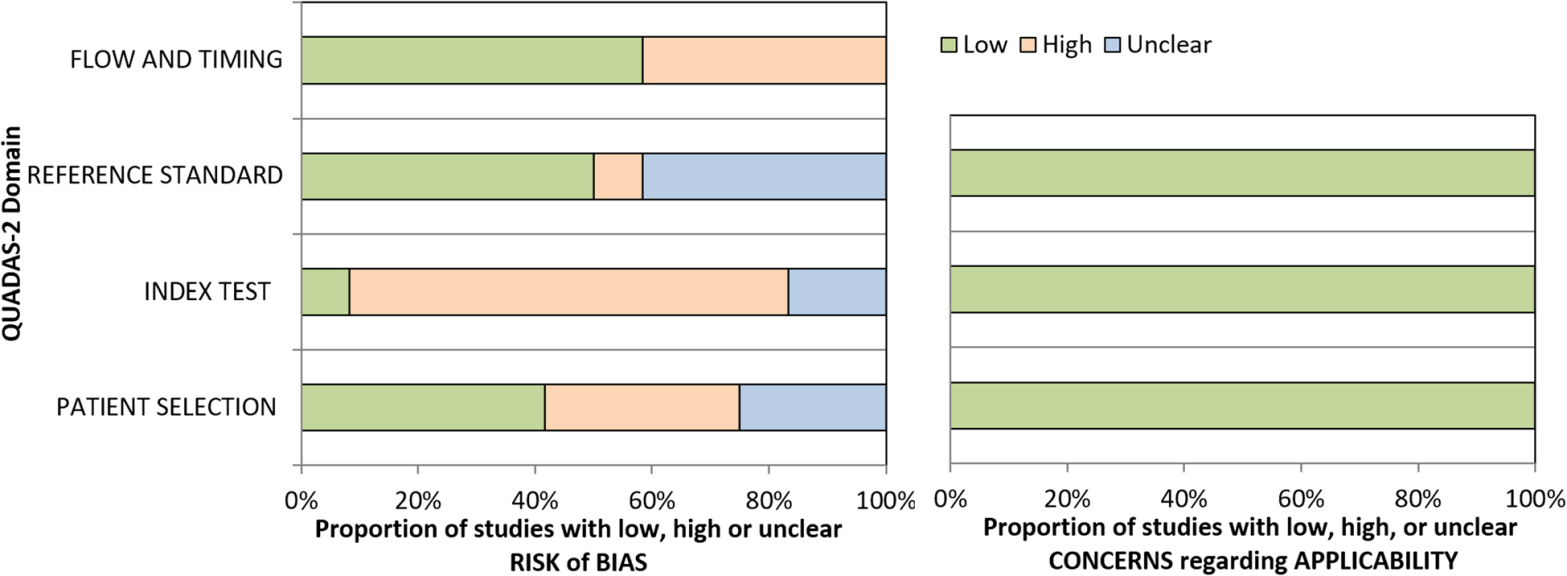
QUADAS-2 quality assessment of selected studies summary and graphical display

All selected studies where assessed on their methodological quality and the risk of bias using the QUADAS-2 tool. A summary of those results is shown in Table 2 and Fig. 2. There were no concerns regarding applicability in all selected studies. However, there was a high risk of bias in several studies on patient selection, index test and/or flow and timing. Some studies did not report sufficient data on patient selection, index test and/or reference standard and therefore these items were scored as an unclear risk of bias. The QUADAS-2 tool does not contain a total score of bias, but the overall quality of the included studies appears to be moderate.

**Table 2:**
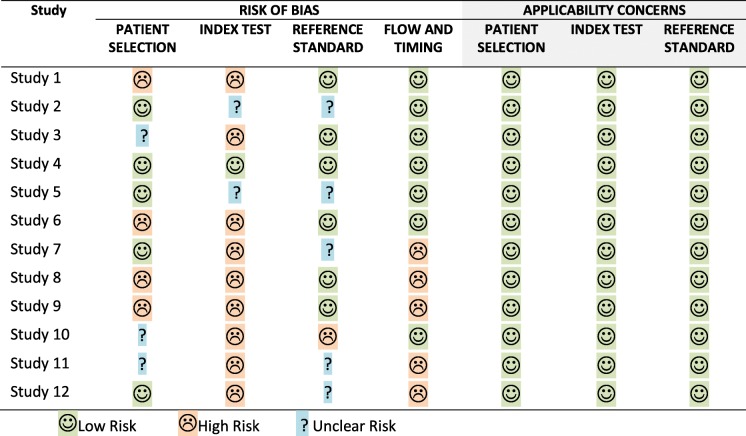
QUADAS-2 quality assessment of selected studies tabular presentation

### Heterogeneity

The part of heterogeneity which was due to the threshold effect was calculated with the Spearman rank correlation coefficient. The spearman correlation coefficient for the three studies on EOS was − 0.500 which indicates that there is a moderate possibility of threshold effect present. The Spearman correlation coefficient for (combined) neonatal sepsis (in total ten studies) was − 0.006, which indicates that there is a low possibility of threshold effect. Because of the low number of studies (two) it was not possible to calculate a spearman correlation coefficient for the LOS group. The inconsistency index of the included studies was between 94.5 and 96.0% for the sensitivity and 0.0 to 92.5% for specificity, which indicates that there was considerable heterogeneity among the included studies.

### Findings

**Fig. 3:**
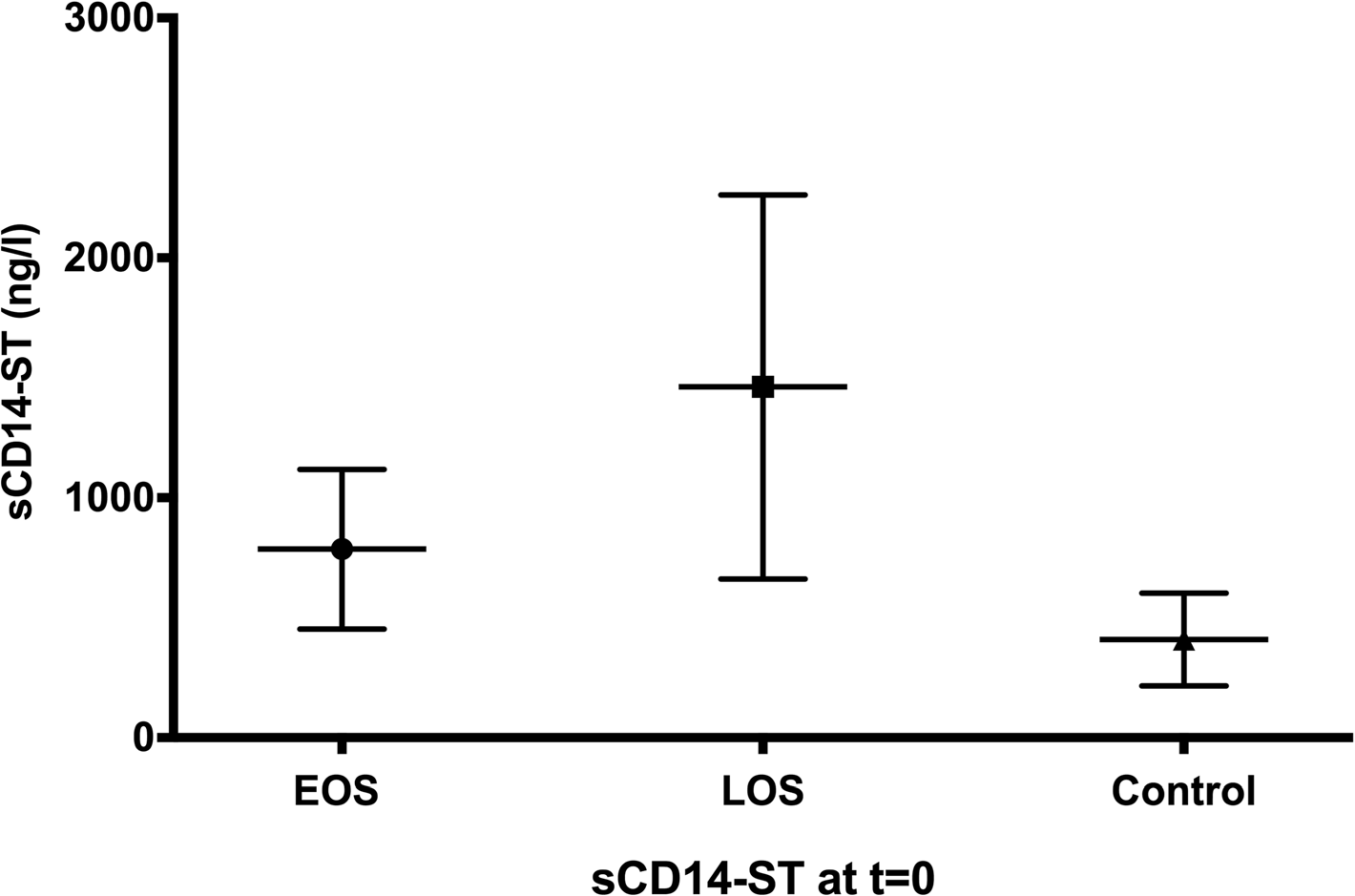
Pooled mean and SD of sCD14-ST levels for EOS, LOS and healthy controls at t = 0. EOS = early-onset sepsis (*n* = 106), LOS = late-onset sepsis (*n* = 65), healthy controls (*n* = 285)

**Fig. 4:**
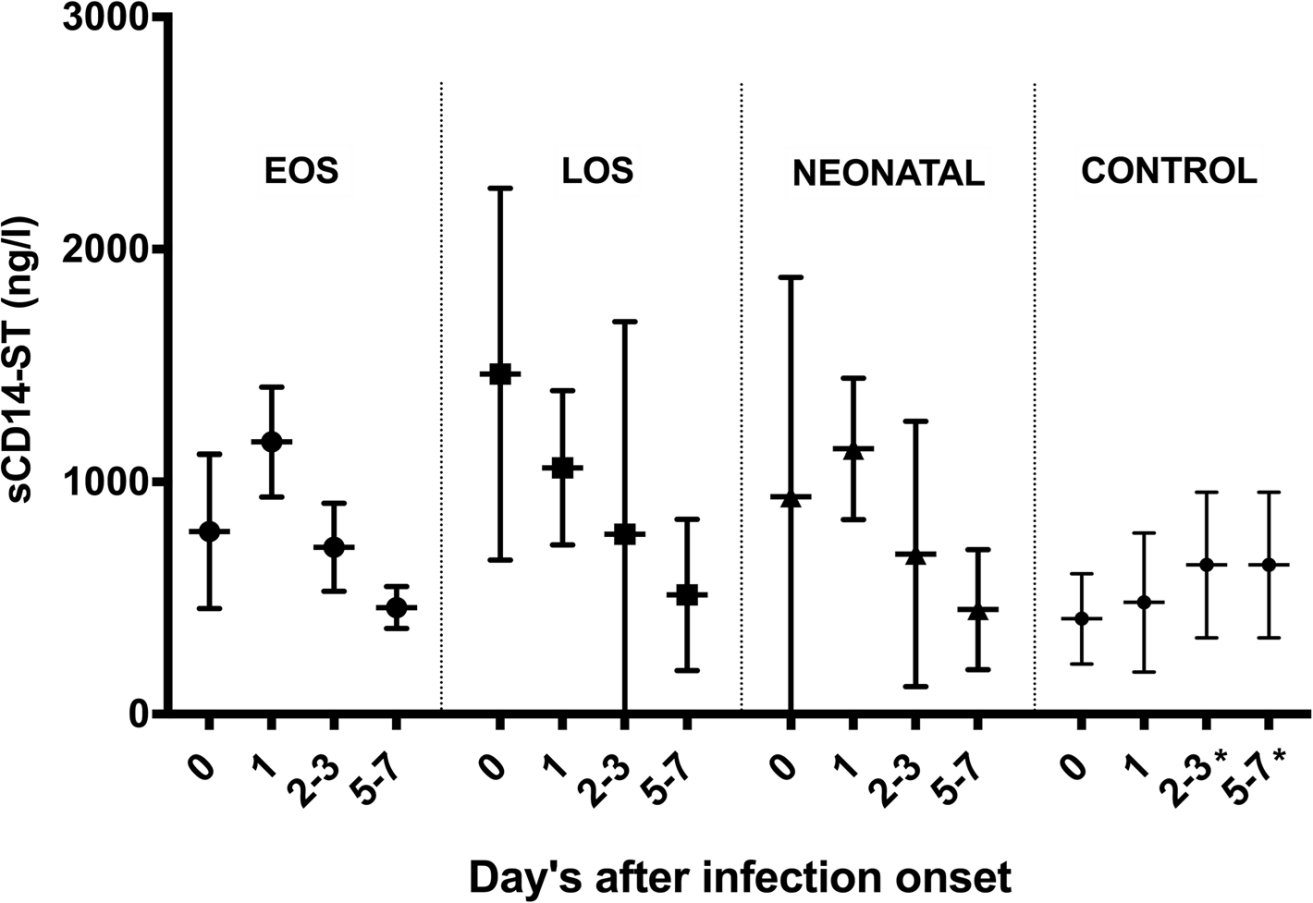
Pooled mean and SD of sCD14-ST levels of each subgroup at different timepoints. EOS = early onset sepsis, LOS = late onset sepsis, Neonatal = neonatal sepsis. * Time intervals 2–3 and 5–7 were taken together

In total 12 studies were used for the qualitative analysis of neonatal sepsis and ten studies in the meta-analysis [9–13, 27, 28, 30, 32, 33]. The included studies analysed sCD14-ST at different time points after sepsis onset. If possible these time points were clustered together in 4 groups for the pooled analysis: t = 0, t = 1, t = 2–3, t = 5–7 (day’s after sepsis onset). The primary outcomes of the included studies are summarized in Additional file 3. The pooled means at t = 0 of EOS cases, LOS cases and healthy controls were unequal according to a one-way ANOVA, F(2, 453) = 219.7, *p* < .0001. Pairwise comparisons of the means using Tukey’s multiple comparisons test indicated a significant difference in all comparisons at t = 0 (Fig. 3 and Table 3). At the other time-points (t = 1, 2–3, 5–7) no significant differences were seen between EOS and LOS. The difference in serum level of sCD14-ST between EOS, LOS, neonatal sepsis and healthy controls is illustrated in Fig. 4. In the first 24 h after sepsis onset a significant increase in pooled means of plasma sCD14-ST levels was seen in EOS (t(71.6) = 7.3, *p* < .0001) while this was not seen in LOS, neonatal sepsis or healthy controls. The original data extracted from the studies on basis of which the calculation of the pool means is performed are reflected in Additional file 4.

**Table 3:** Pooled means and the differences per group at t = 0

	Mean 1(SD)	Mean 2 (SD)	Mean Diff	SE of diff	n1	n2	q	df	Adjusted *p* value
EOS vs. LOS	786 (332)	1463 (800)	− 677	58.85	106	65	16.27	453	<.0001
EOS vs. Control	786 (332)	410 (194)	376	42.5	106	285	12.51	453	<.0001
LOS vs. Control	1463 (800)	410 (194)	1053	51.35	65	285	29	453	<.0001

*df* degrees of freedom, *EOS* early-onset sepsis, *LOS* late-onset sepsis, *SE* standard error; Studies included for this analysis: EOS *n* = 4 [9, 11, 27, 28]; LOS *n* = 3 [10, 12, 27]; Controls *n* = 9 [9–13, 27, 28, 30, 32]

### Early-onset sepsis

**Fig. 5:**
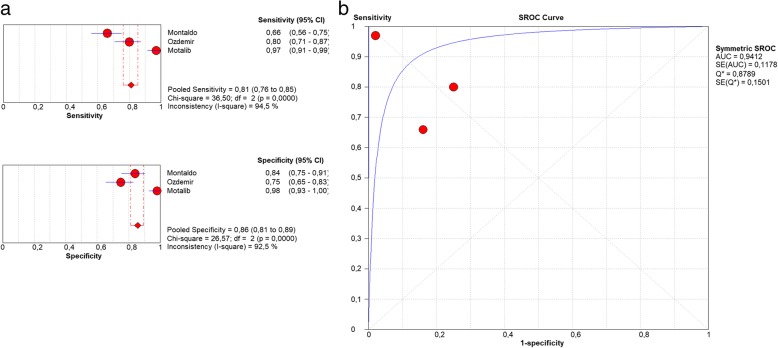
**a** Forest plot of sensitivity and specificity of sCD14-ST levels in EOS at t = 0. **b** Summary ROC plot of sensitivity and specificity of sCD14-ST for EOS at t = 0. EOS = early onset sepsis

Optimal cut-off values at t = 0 ranged from 305 to 672 ng/l for EOS cases versus healthy controls. The pooled sensitivity was 81% (95%CI: 0.76–0.85), the pooled specificity was 86% (0.81–0.89) with an AUC of 0.9412 (SE 0.1178) (Fig. 5).

### Late-onset sepsis

**Fig. 6:**
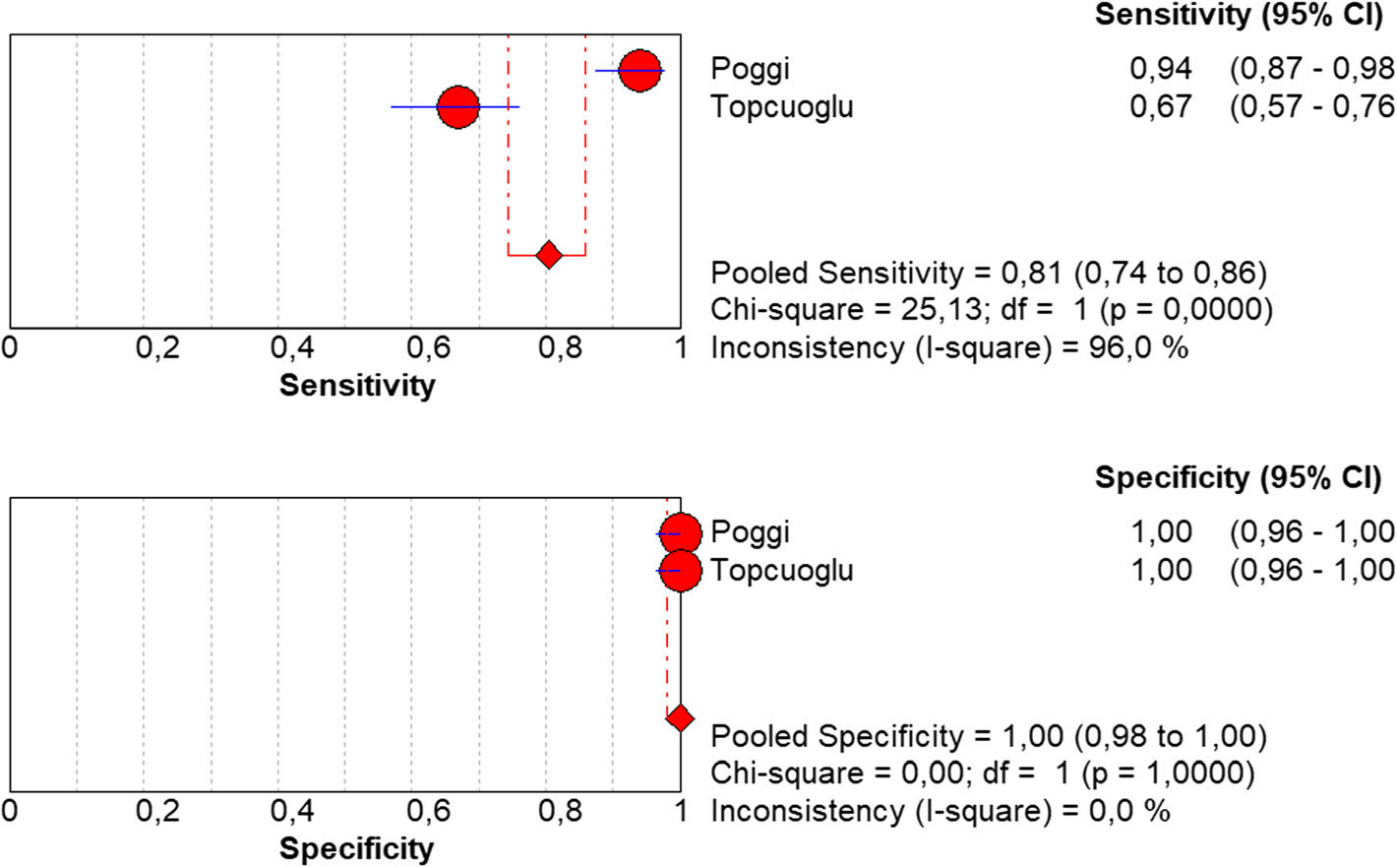
Forest plot of sensitivity and specificity of sCD14-ST levels in LOS at t = 0. LOS = late onset sepsis

Optimal cut-off values at t = 0 ranged from 801 to 885 ng/l for LOS cases versus healthy controls. The pooled sensitivity was 81% (0.74–0.86) and the pooled specificity was 100% (0.98–1.00) (Fig. 6). An AUC and SROC was not estimable because of the low number of studies.

### Neonatal sepsis

**Fig. 7:**
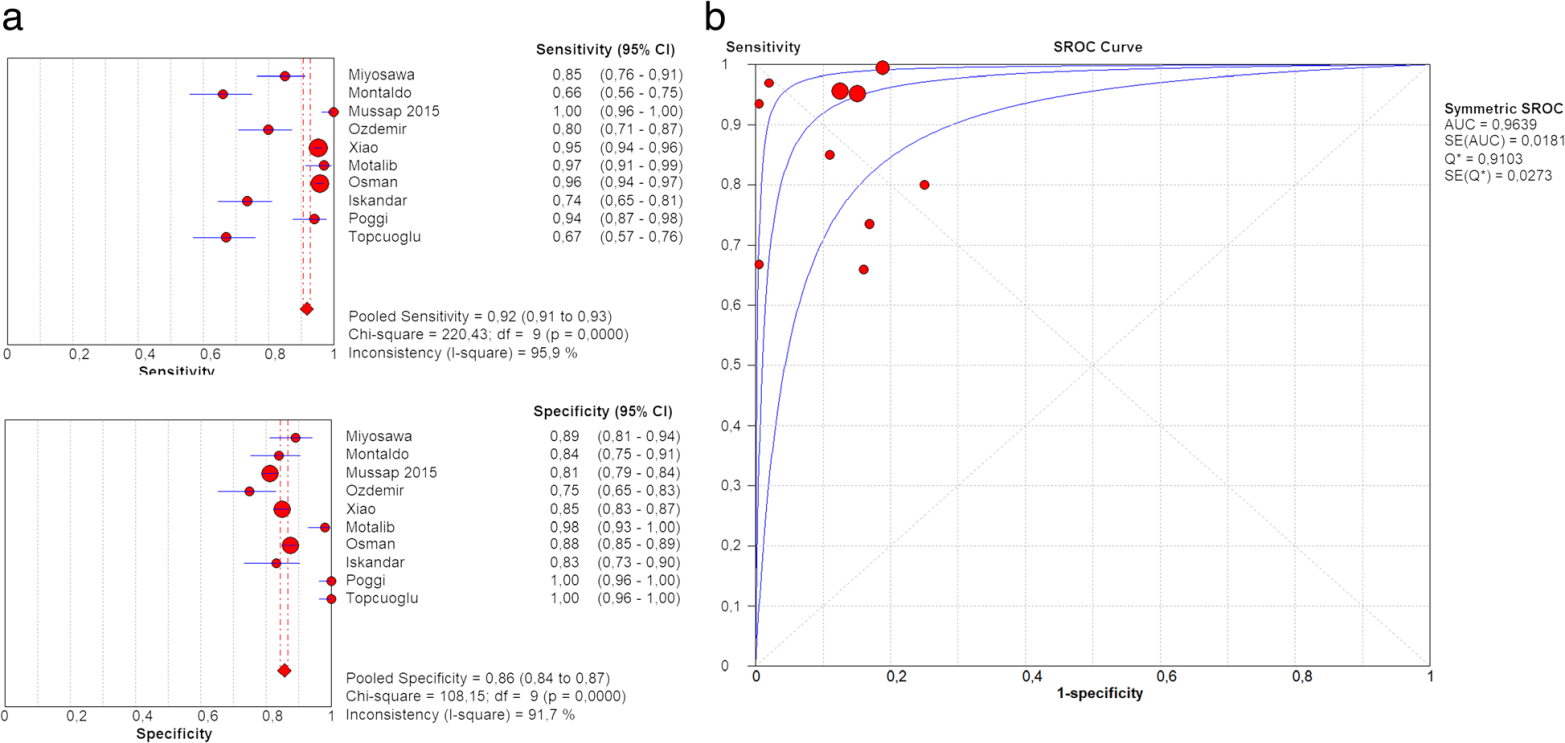
**a** Forest plot of sensitivity and specificity of sCD14-ST levels in neonatal sepsis at t = 0. **b** Summary ROC plot of sensitivity and specificity of sCD14-ST for neonatal sepsis at t = 0

Taken all sepsis cases together (EOS, LOS and combined), cut-off values ranged from 305 to 885 ng/l. The pooled sensitivity was 92% (0.91–0.93) and the pooled specificity was 86% (0.84–0.87) with an AUC of 0.9639 (0.0181) (Fig. 7).

## Discussion

In this systematic review and meta-analysis, the current literature of sCD14-ST as biomarker for neonatal sepsis has been evaluated in order to facilitate rapid diagnosis and reduce the use of antibiotics in neonates. At sepsis onset a consequently higher level of sCD14-ST was found in septic neonates compared to healthy controls with higher levels in LOS compared to EOS. sCD14-ST levels increase in the first 24 h in EOS but not in LOS. Furthermore a decrease in sCD14-ST levels is seen in the days after treatment initiation for both EOS and LOS.

Our findings are in line with the promising results in adult research, were sCD14-ST has been proven to be a specific and quick-responding biomarker for bacterial infections [34]. In contrast to CRP and PCT, sCD14-ST is very specific for bacteria. Moreover sCD14-ST seems to be fast and more rapid-responding then CRP (increases after 12–24 h) and also PCT (increases after 3–4 h) with plasma levels already increased at sepsis onset (t = 0) [6, 35]. Although PCT seems promising in reducing the duration of antibiotic therapy after the initial start of the antibiotic treatment, there is no added value in the decision making before start of the antibiotics at t = 0 [36].

In the studies presented in this systematic review, differences in sCD14-ST levels were seen between EOS and LOS. This underlines the importance to consider EOS and LOS as two different disease entities, requiring separate analysis in original articles and systematic reviews. The hypothesis is that lower levels and increase in the first 24 h of sCD14-ST in EOS are caused by the fact that sepsis evaluation in EOS is determined in the early ‘pre-clinical’ stage of the infection, whereas sCD14-ST in LOS is analysed in the clinical stage with the first symptoms of infection. This finding, assuming a time-dependency of sCD14-ST, is in line with the decrease in sCD14-ST levels after initiation of therapy in both EOS and LOS. The change of sCD14-ST over time might be of future clinical value and can be used in both diagnosis and efficacy of treatment of neonatal sepsis.

The cut-off values, together with the specificity and sensitivity varied greatly between the studies and were depended on the type of infection (EOS vs LOS) and sampling time. In future practice, a cut-off value with a near 100% sensitivity or a determined amount of measurements is required to rule out the possibility of neonatal sepsis. Hence, a cut-off value of sCD14-ST for both EOS and LOS to rule out neonatal sepsis has yet to be determined, because most included studies have chosen a higher specificity over a higher sensitivity.

The literature search resulted in 12 articles covering the subject, indicating that this is a relatively new area of interest. Using the QUADAS-2 tool, the overall quality of the selected studies appears to be moderate. Importantly, with the QUADAS-2 tool we estimated there to be a high risk of bias on the thresholds of the index test in our included studies, because this was not pre-specified. However this result was distorted, as those studies were performed just to determine reference ranges.

This review has some limitations. First, the included studies used different reference standards to define neonatal sepsis, varying from blood culture to a combination of clinical signs. Although bacterial confirmation is the golden standard diagnostic test, it has a high-false negative rate in septic newborns [37]. Second, there were two PATHFAST measuring instruments from two different companies used in the included studies (Mitsubishi Chemical Medience Corporation from Tokyo, Japan & Gepa Diagnostics from Milan, Italy). A comparative study between the instruments produced by the different companies has not been performed, therefore the influence on the outcome of the sCD14-ST level is unclear. Third, most of the studies did not differentiate between EOS and LOS, making it difficult to analyse the two disease entities separately. Furthermore, the sample size in most of the included studies was small. Lastly, despite the possibility of a threshold effect in the LOS studies, we decided to perform a pooled accuracy analysis, therefore this should be interpreted with caution.

## Conclusions

Regarding this meta-analysis we can conclude that sCD14-ST is a promising diagnostic biomarker for EOS and LOS. The difference in pooled means between EOS and LOS underlines the importance to consider EOS and LOS as two different disease entities, in which bloodsamples in EOS are measured in the early ‘pre-clinical’ stage of the infection and in LOS bloodsamples are measured in the clinical stage of infection. Therefore EOS and LOS requiring separate analysis in original articles and systematic reviews. In future daily practice sCD14-ST might be a valuable biomarker to rule out EOS prior to treatment initiation. Although a clear cut-off value with a high negative predictive value has still to be determined and evaluated in future prospective studies. Future studies should use a larger number of patients in their research populations. Lastly, more research on serial measurements of sCD14-ST in the first 24 h after birth is necessary for better understanding the role of sCD14-ST in EOS.

### Additional files

Additional file 1: Prisma 2009 checklist. (DOC 65 kb)

Additional file 2: Logbook of literature search. (DOCX 36 kb)

Additional file 3: Primary outcomes of the included studies. (DOCX 19 kb)

Additional file 4: Original data of the included studies. (DOCX 21 kb)

## Data Availability

All data analysed during this study are included in this published article (and its supplementary information files) and are available from the included studies, which are fully referenced.

## Appendix

### Data synthesis

When median (md), minimum (a) and maximum (b) were reported, mean (m) and standard deviation (SD or S) were calculated based on the formulas of Hozo et al. [19] For samples sizes smaller than 25, the estimated mean was calculated with formula 1 = $\mathrm{m}=\frac{\left(\mathrm{a}+2\ \mathrm{x}\ \mathrm{m}+\mathrm{b}\right)}{4}$. For samples sizes larger than 25, the mean was estimated as the median (formula 2). The standard deviation for samples sizes smaller than 15 were calculated as based on formula 3 = ${\mathrm{S}}^2=\frac{1}{12}\left(\frac{{\left(\mathrm{a}-2\mathrm{md}+\mathrm{b}\right)}^2}{4}\right)+{\left(\mathrm{b}-\mathrm{a}\right)}^2$. When sample sizes are between 16 and 70 formula 4 (= range/4) was used. When the median and interquartile range (q1-q3) were given calculations were based on a study of Wan and colleagues. [18] Formula 5 was applied to estimate the mean ((q1 + median + q3)/3). In this case the standard deviation was calculated using formula 6 = (q3-q1) / 1,35. The pooled mean was calculated by the formula $\mathrm{m}=\frac{\left(\mathrm{n}1\ \mathrm{x}\ \mathrm{m}1+\mathrm{n}2\ \mathrm{x}\ \mathrm{m}2\right)}{\mathrm{n}1+\mathrm{n}2}$ and the pooled standard deviation was calculated by the formula $Var=\frac{\left(\mathrm{n}1\ \mathrm{x}\ \mathrm{Var}1+\mathrm{n}2\ \mathrm{x}\ \mathrm{Var}2+\mathrm{n}1\ \mathrm{c}\ {\left(\mathrm{m}1-\mathrm{m}\right)}^2+\mathrm{n}2\ \mathrm{x}\ {\left(\mathrm{m}2-\mathrm{m}\right)}^2\right)}{\mathrm{n}1+\mathrm{n}2}$, in which standard deviation equals the square root of the variance.

## Abbreviations

95%CI: 95% confidence interval
ANOVA: Analysis of variance
AUC: Area under the curve
CLEIA: Chemiluminescent enzyme immunoassay
CRP: C-reactive protein
CTC: Clinical Trials in Children
EOS: Early onset sepsis
IQR: Interquartile range
I-squared: Inconsistency index
LBW: Low birth weight
LOS: Late onset sepsis
LPS: Lipopolysaccharides
NPV: Negative predicted value
PCT: procalcitonin
PPV: Positive predicted value
PRISMA: Preferred Reporting Items for Systematic Reviews and Meta-analyses Statement
P-SEP: Presepsin
QUADAS-2: Quality Assessment of Diagnostic Accuracy Studies 2
sCD14-ST: Soluble cluster of differentiation 14 subtype
SD: Standard deviation
SE: Standard error
SROC: Summary ROC
VLBW: Very low birth weight
