# Supplementary material for: Soluble CD14 subtype (sCD14-ST) as biomarker in neonatal early-onset sepsis and late-onset sepsis: a systematic review and meta-analysis
Source: BMC Immunol. 2019 Jun 3;20:17. doi: 10.1186/s12865-019-0298-8 (PMC6547508; doi:10.1186/s12865-019-0298-8)
Supplement: Supplementary file 2 — Logbook of literature search. (DOCX 36 kb) [file 12865_2019_298_MOESM2_ESM.docx]

**APPENDIX B – LOGBOOK OF LITERATURE SEARCH**

Content

[PubMed, 16-03-2017 1](#_Toc477767657)

[Embase.com, 16-03-2017 2](#_Toc477767658)

[The Cochrane Library, 16-03-2017 2](#_Toc477767659)

[Web of Science, 16-03-2017 3](#_Toc477767660)

[WHO ICTRP Clinical Trials in Children (CTC), 16-03-2017 3](#_Toc477767662)

[Clinicaltrials.gov, 16-03-2017 3](#_Toc477767663)

**Limits (e.g. Languages, Publication dates):** none

**Search terms**

Presepsin[tiab] OR sCD14[tiab] OR sCD14-ST[tiab] OR soluble CD14[tiab] OR P-SEP[tiab]

“Infant, Newborn”[MeSH] OR newborn*[tiab] OR prematur*[tiab] OR low birth weight[tiab] OR VLBW[tiab] OR LBW[tiab] or infant*[tiab] or neonat*[tiab] OR postmatur*[tiab] OR preterm*[tiab] OR new-born*[tiab] OR neo-nat*[tiab]

| **Database** | **Before duplicates removed** | **After duplicates removed** |
| --- | --- | --- |
| PubMed | 95 |  |
| Embase | 121 |  |
| CENTRAL | 23 |  |
| Web of Science | 201 |  |
| WHO ICTRP CTC | 14 |  |
| Clinicaltrials.gov | 22 |  |
| **Total** | 476 | 290 |

# PubMed, 16-03-2017

Keyword RefMan: PM06032017

| Search | Query | Items found |
| --- | --- | --- |
| [#6](https://www.ncbi.nlm.nih.gov/pubmed/advanced) | Search **(("presepsin protein, human" [Supplementary Concept] OR Presepsin[tiab] OR sCD14[tiab] OR sCD14-ST[tiab] OR soluble CD14[tiab] OR P-SEP[tiab])) AND (“Infant, Newborn”[MeSH] OR newborn*[tiab] OR prematur*[tiab] OR low birth weight[tiab] OR VLBW[tiab] OR LBW[tiab] or infant*[tiab] or neonat*[tiab] OR postmatur*[tiab] OR preterm*[tiab] OR new-born*[tiab] OR neo-nat*[tiab])** | [95](https://www.ncbi.nlm.nih.gov/pubmed/?cmd=HistorySearch&querykey=6) |
| [#5](https://www.ncbi.nlm.nih.gov/pubmed/advanced) | Search **“Infant, Newborn”[MeSH] OR newborn*[tiab] OR prematur*[tiab] OR low birth weight[tiab] OR VLBW[tiab] OR LBW[tiab] or infant*[tiab] or neonat*[tiab] OR postmatur*[tiab] OR preterm*[tiab] OR new-born*[tiab] OR neo-nat*[tiab]** | [978392](https://www.ncbi.nlm.nih.gov/pubmed/?cmd=HistorySearch&querykey=5) |
| [#1](https://www.ncbi.nlm.nih.gov/pubmed/advanced) | Search **"presepsin protein, human" [Supplementary Concept] OR Presepsin[tiab] OR sCD14[tiab] OR sCD14-ST[tiab] OR soluble CD14[tiab] OR P-SEP[tiab]** | [1330](https://www.ncbi.nlm.nih.gov/pubmed/?cmd=HistorySearch&querykey=1) |

# Embase.com, 16-03-2017

Keyword RefMan: EM16032017

| No. | Query | Results |
| --- | --- | --- |
| #3 | **#1** AND **#2** | **121** |
| #2 | **presepsin**:ab,ti OR **scd14**:ab,ti OR **'scd14 st'**:ab,ti OR **'soluble cd14'**:ab,ti OR **'p sep'**:ab,ti | **1912** |
| #1 | **'newborn'**/exp OR **'prematurity'**/exp OR **'low birth weight'**/exp OR **'postmaturity'**/exp OR **newborn***:ab,ti OR **prematur***:ab,ti OR **'low birth weight'**:ab,ti OR **vlbw**:ab,ti OR **lbw**:ab,ti OR **infant***:ab,ti OR **neonat***:ab,ti OR **postmatur***:ab,ti OR **preterm***:ab,ti OR **'new born*'**:ab,ti OR **'neo nat*'**:ab,ti | **1094141** |

# The Cochrane Library, 16-03-2017

**Keyword RefMan: CE16032017**

| ID | Search | Hits |
| --- | --- | --- |
| #1 | newborn* or prematur* or 'low birth weight' or vlbw or lbw or infant* or neonat* or postmatur* or preterm* or 'new born*' or 'neo nat*':ti,ab,kw (Word variations have been searched) | 59727 |
| #2 | presepsin or scd14 or 'scd14 st' or 'soluble cd14' or 'p sep':ti,ab,kw (Word variations have been searched) | 484 |
| #3 | #1 and #2 | 25 |

All Results (25)

Cochrane Reviews (2) All Review Protocol

Other Reviews (0) Trials (23) Methods Studies (0) Technology Assessments (0) Economic Evaluations (0) Cochrane Groups (0)

# Web of Science, 16-03-2017

Keyword RefMan: WOS16032017

All Databases: Web of ScienceTM Core CollectionKCI-Korean Journal DatabaseMEDLINE®Russian Science Citation IndexSciELO Citation Index


| **Set** | **Results** |  |
| --- | --- | --- |
| # 1 | [**201**](http://apps.webofknowledge.com/summary.do?product=WOS&doc=1&qid=8&SID=Y21l5gJo8bKWEQsHXuj&search_mode=GeneralSearch&update_back2search_link_param=yes) | **TOPIC:** ((presepsin OR scd14 OR scd14 st OR soluble cd14 OR p sep)) *AND* **TOPIC:** ((newborn* or prematur* or “low birth weight” or vlbw or lbw or infant* or neonat* or postmatur* orpreterm* or “new born*” or “neo nat*”))  *Indexes=SCI-EXPANDED, SSCI, A&HCI, ESCI Timespan=All years* |

# WHO ICTRP Clinical Trials in Children (CTC), 16-03-2017

Keyword RefMan: IC16032017

14 records for 14 trials found for: presepsin OR scd14 OR scd14 st OR soluble cd14 OR p sep

# Clinicaltrials.gov, 16-03-2017

Keyword RefMan: CT16032017

**22 studies found for**:    presepsin OR scd14 OR 'scd14 st' OR 'soluble cd14' OR 'p sep' | Child
