# Supplementary material for: Soluble CD14 subtype (sCD14-ST) as biomarker in neonatal early-onset sepsis and late-onset sepsis: a systematic review and meta-analysis
Source: BMC Immunol. 2019 Jun 3;20:17. doi: 10.1186/s12865-019-0298-8 (PMC6547508; doi:10.1186/s12865-019-0298-8)
Supplement: Supplementary file 3 — Primary outcomes of the included studies. (DOCX 19 kb) [file 12865_2019_298_MOESM3_ESM.docx]

**APPENDIX D – PRIMARY OUTCOMES OF THE INCLUDED STUDIES**

| Study | Patients (n) | Time (days) | | Cut-off value* | AUC | Sensitivity | Specificity | PPV | NPV | Median (IQR range)^#^ | Mean ± SD^#^ |
| --- | --- | --- | --- | --- | --- | --- | --- | --- | --- | --- | --- |
| Early onset sepsis |  |  | |  |  |  |  |  |  |  |  |
| Montaldo | 32 | T = 0 | | 453 | 0.75 | 66% | 84% | 82% | 65% | P: 598 (457-787)  C: 328 (311-527) |  |
|  |  | T = 1/2 | | 653 | 0.92 | 88% | 94% | 93% | 89% | P: 802 (511-1006)  C: 385 (280-587) |  |
|  |  | T = 1 | | 788 | 0.97 | 93% | 100% | 100% | 94% | P: 1228 (738-1546)  C: 504 (212-646) |  |
|  |  | T = 2 | | 744 | 0.90 | 79% | 92% | 85% | 87% | P: 979 (588-1031)  C: 476 (227-602) |  |
| Ozdemir | 29 | T = 0 | | 539 | 0.77 | 80% | 75% | 91% | 59% | P: 651 (436-1159)  C: 498 (316-882) | P: 704 ± 224  C: 508 ± 165 |
|  |  | T = 3 | |  |  |  |  |  |  |  | P: 554 ± 144 |
|  |  | T = 7 | |  |  |  |  |  |  |  | P: 458 ± 91 |
|  |  | T = 5 | |  |  |  |  |  |  | P: 360 (293-427) |  |
| Motalib | 28 | T = 0 | | 672 | 0.95 | 97% | 98% | 96% | 92% |  | P: 873 ± 234  C: 380 ± 127 |
|  |  | T = 7 | |  |  |  |  |  |  |  | P: 325 ± 87 |
| Combined | |  |  |  |  |  |  |  |  |  |  |
| Miyosawa | 13 | T = 0 | | 795 | 0.87 | 85% | 89% | 85% | 89% | P: 953 (708-1381)  C: 596 (542-714) |  |
|  |  | T = 1 | |  |  |  |  |  |  | P: 1167 (881-1643)  C: 571 (548-619) |  |
|  |  | T = 2 | |  |  |  |  |  |  | P: 1107 (798-1690)  C: 667 (548-786) |  |
| Mussap (2015) | 25 | T = 1-7 | | 548  600 | 0.99 | 100%  98% | 81%  100% |  |  | P: 1000 (862-1212)  C: 453 (309-526) |  |
| Osman | 40 | T = 0 | | 875 | 0.95 | 96% | 88% |  |  |  | P: 1176 ± 444  C: 550 ± 76 |
| Iskandar | 35 | T = 0 | | 707 | 0.76 | 86% | 69% | 86% | 69% |  |  |
| Xiao  (hematosepsis) | 42 | T = 0 | | 305 | 0.94 | 95% | 85% |  |  | P: 786 (675-897)  C: 124 (114-135) |  |
|  |  | T = 3 | |  |  |  |  |  |  | P: 533 (451-615) |  |
| Xiao  (non-hematosepsis) | 54 | T = 0 | |  |  |  |  |  |  | P: 626 (556-696) |  |
| Late onset sepsis | |  |  |  |  |  |  |  |  |  |  |
| Poggi | 19 | T = 0 | | 885 | 0.97 | 94% | 100% | 100% | 95% | P: 1295 (977-1500)  C: 562 (337-726) |  |
|  |  | T = 1 | |  |  |  |  |  |  | P: 1011 (861-1309)  C: 481 (310-704) |  |
|  |  | T = 3 | |  |  |  |  |  |  | P: 968 (538-1344)  C: 459 (302-622) |  |
|  |  | T = 5 | |  |  |  |  |  |  | P: 889 (388-1031)  C: 422 (291-509) |  |
| Topcuoglu | 42 | T = 0 | | 801 | 0.86 | 67% | 100% | 100% | 74% | P: 1024  C: 530 (363-580) |  |
|  |  | T = 2 | |  |  |  |  |  |  | P: 711 |  |
|  |  | T = 6 | |  |  |  |  |  |  | C: 442 |  |
| Healthy controls |  |  | |  |  |  |  |  |  |  |  |
| Mussap (2012) | 26 | T = 0 | |  |  |  |  |  |  | C: 578 (453-796) | C: 643 ± 304 |
| Pugni | 684 | T = 3-4 | |  |  |  |  |  |  | C: 604 (467-791) | C: 649 ± 257 |
|  |  | T = 3-7 | |  |  |  |  |  |  | C: 620 (503-864) | C: 720 ± 329 |

# P = Patients (septic neonates), C = Controls (healthy neonates)

* All sCD14-ST values are reported in ng/l
