# Supplementary material for: Soluble CD14 subtype (sCD14-ST) as biomarker in neonatal early-onset sepsis and late-onset sepsis: a systematic review and meta-analysis
Source: BMC Immunol. 2019 Jun 3;20:17. doi: 10.1186/s12865-019-0298-8 (PMC6547508; doi:10.1186/s12865-019-0298-8)
Supplement: Supplementary file 4 — Original data of the included studies. (DOCX 21 kb) [file 12865_2019_298_MOESM4_ESM.docx]

**APPENDIX E – ORIGINAL DATA OF THE INCLUDED STUDIES**

| **EARLY ONSET SEPSIS** | | | | | | | | | | | |
| --- | --- | --- | --- | --- | --- | --- | --- | --- | --- | --- | --- |
|  | **t (days)** | **n** | **subgroup^a^** | **median** | **IQR low** | **IQR upp** | **min** | **max** | **mean** | **SD** | **formulas^b^** |
| Montaldo | t=0 | 32 | culture-pos | 598 | 457 | 787 | NR | NR | *614* | *244* | 5,6 |
|  | t=1/2 | 32 | culture-pos | 802 | 511 | 1006 | NR | NR | *773* | *151* | 5,6 |
|  | t=1 | 32 | culture-pos | 1228 | 738 | 1546 | NR | NR | *1171* | *236* | 5,6 |
|  | t=2 | 32 | culture-pos | 979 | 588 | 1031 | NR | NR | *866* | *39* | 5,6 |
| Osman | t=0 | 17 | combined | NR | NR | NR | NR | NR | 1110 | 462 | n/a |
| Ozdemir | t=0 | 29 | combined | NR | NR | NR | NR | NR | 704 | 224 | n/a |
|  | t=3 | 29 | combined | NR | NR | NR | NR | NR | 554 | 144 | n/a |
|  | t=7 | 29 | combined | NR | NR | NR | NR | NR | 458 | 91 | n/a |
| Xiao | t=0 | 42 | culture-pos | 786 | 675 | 897 | *171* | *1439* | *786* | *317* | 2,4 |
|  | t=0 | 54 | probable | 626 | 556 | 969 | *241* | *1069* | *626* | *207* | 2,4 |
|  | t=3 | 42 | culture-pos | 533 | 451 | 615 | NR | NR | *533* | *121* | 5,6 |
|  | t=5 | 42 | culture-pos | 360 | 293 | 427 | NR | NR | *360* | *99* | 5,6 |
| Motalib | t=0 | 28 | combined | NR | NR | NR | NR | NR | 873 | 234 | n/a |
|  |  |  |  |  |  |  |  |  |  |  |  |
| **LATE ONSET SEPSIS** | | | | | | | |  |  |  |  |
|  | **t (days)** | **n** | **subgroup^a^** | **median** | **IQR low** | **IQR upp** | **min** | **max** | **mean** | **SD** | **formulas^b^** |
| Osman | t=0 | 23 | combined | NR | NR | NR | NR | NR | 1225 | 434 | n/a |
| Poggi | t=0 | 19 | combined | 1295 | 977 | 1500 | NR | NR | *1257* | *387* | 5,6 |
|  | t=1 | 19 | combined | 1011 | 861 | 1309 | NR | NR | *1060* | *332* | 5,6 |
|  | t=3 | 15 | combined | 968 | 538 | 1344 | NR | NR | *950* | *597* | 5,6 |
|  | t=5 | 15 | combined | 889 | 388 | 1031 | NR | NR | *769* | *476* | 5,6 |
| Topcuoglu | t=0 | 19 | probable | 1033 | NR | NR | 398 | 8202 | *2667* | *1951* | 1,4 |
|  | t=0 | 23 | culture-pos | 1200 | NR | NR | 295 | 4785 | *1870* | *1123* | 1,4 |
|  | t=0 | 42 | combined | 1024 | NR | NR | 295 | 8202 | *1024* | *1977* | 2,4 |
|  | t=3 | 42 | combined | 711 | NR | NR | 213 | 4200 | *711* | *997* | 2,4 |
|  | t=7 | 42 | combined | 422 | NR | NR | 199 | 901 | *422* | *176* | 2,4 |
|  |  |  |  |  |  |  |  |  |  |  |  |
| **HEALTHY CONTROLS** | | | | | |  |  |  |  |  |  |
|  | **t (days)** | **n** | **subgroup^a^** | **median** | **IQR low** | **IQR upp** | **min** | **max** | **mean** | **SD** | **formulas^b^** |
| Montaldo | t=0 | 38 | n/a | 328 | 311 | 527 | NR | NR | *389* | *160* | 5,6 |
|  | t=1/2 | 38 | n/a | 385 | 280 | 587 | NR | NR | *417* | *227* | 5,6 |
|  | t=1 | 38 | n/a | 504 | 212 | 646 | NR | NR | *454* | *321* | 5,6 |
|  | t=2 | 38 | n/a | 476 | 227 | 602 | NR | NR | *383* | *278* | 5,6 |
| Mussap (2012) | t=1-7 | 26 | n/a | 578 | 453 | 796 | 255 | 1144 | 643 | 304 | 2,4 |
| Mussap (2015) | t=0 | 32 | n/a | 453 | 309 | 526 | *214* | *603* | 453 | 97 | 2,4 |
| Miyosawa | t=0 | 12 | n/a | 596 | 542 | 714 | *380* | *774* | *586* | *114* | 1,3 |
|  | t=1 | 6 | n/a | 571 | 548 | 619 | *541* | *634* | *579* | *27* | 1,3 |
|  | t=2 | 8 | n/a | 667 | 548 | 786 | *417* | *799* | *638* | *112* | 1,3 |
| Motalib | t=0 | 34 | n/a | NR | NR | NR | NR | NR | 380 | 127 | n/a |
| Osman | t=0 | 15 | n/a | NR | NR | NR | NR | NR | 550 | 76 | n/a |
| Ozdemir | t=0 | 40 | n/a | NR | NR | NR | NR | NR | 508 | 165 | n/a |
| Poggi | t=0 | 21 | n/a | 562 | 337 | 726 | NR | NR | *542* | *288* | 5,6 |
|  | t=1 | 21 | n/a | 481 | 310 | 704 | NR | NR | *498* | *292* | 5,6 |
|  | t=3 | 21 | n/a | 459 | 302 | 622 | NR | NR | *461* | *237* | 5,6 |
|  | t=5 | 21 | n/a | 422 | 291 | 509 | NR | NR | *407* | *161* | 5,6 |
| Pugni | t=3-4^c^ | 484 | n/a | 604 | 466 | 791 | NR | NR | 649 | 257 | n/a |
|  | t=3-7^d^ | 195 | n/a | 620 | 503 | 864 | NR | NR | 720 | 329 | n/a |
| Topcuoglu | t=0 | 40 | n/a | 530 | NR | NR | 190 | 782 | *530* | *148* | 2,4 |
| Xiao | t=0 | 53 | n/a | 124 | 114 | 135 | *55* | *200* | *124* | *36* | 2,4 |
|  |  |  |  |  |  |  |  |  |  |  |  |
| **COMBINED** | | | | | |  |  |  |  |  |  |
|  | | | | | |  |  |  |  |  |  |
|  | **t (days)** | **n** | **subgroup^a^** | **median** | **IQR low** | **IQR upp** | **min** | **max** | **mean** | **SD** | **formulas^b^** |
| Mussap (2015) | t=0 | 40 | culture-pos | 1000 | 862 | 1212 | *555* | *9379* | *1000* | *2206* | 2,4 |
|  | t=0 | 30 | probable | 992 | 737 | 1585 | *410* | *2952* | *992* | *636* | 2,4 |
|  | t=3 | 10 | culture-pos | NR | NR | NR | NR | NR | NR | NR |  |
| Miyosawa | t=0 | 13 | culture-pos | 953 | 708 | 1381 | *400* | *2049* | *1089* | *482* | 1,3 |
|  | t=1 | 11 | culture-pos | 1167 | 881 | 1643 | *547* | *1891* | *1193* | *388* | 1,3 |
|  | t=2 | 12 | culture-pos | 1107 | 798 | 1690 | *333* | *2168* | *1179* | *531* | 1,3 |
| Osman | t=0 | 40 | combined | NR | NR | NR | NR | NR | 1176 | 444 | n/a |
|  | t=0 | 23 | culture-pos | NR | NR | NR | NR | NR | 1454 | 372 | n/a |
|  | t=0 | 17 | probable | NR | NR | NR | NR | NR | 801 | 169 | n/a |

t = time in days; n = number of patients;

numbers in italic and orange reflect numbers that are extracted from figures

numbers in italic and blue reflect numbers that are calculated based on the formulas of the last colum

culture-pos = subgroup consists of patients with a positive blood culture;

^a^ subgroups are divided in ‘culture-pos’ = patients with a positive blood culture, ‘probable’ = patients with clinical symptoms of sepsis but with a negative blood culture, ‘combined’ = patients with clinical symptoms of sepsis with or without a positive blood culture

^b^ the formulas used to calculated the mean and SD depend on the number of patients and the available data, and are described in the methods section of the article.

^c^ gestational age >37wk

^d^ gestational age 24-36wk
